# Morphology and ontogeny of *Lophopus crystallinus* lophophore support the epistome as ancestral character of phylactolaemate bryozoans

**DOI:** 10.1007/s00435-018-0402-2

**Published:** 2018-03-16

**Authors:** Thomas Schwaha

**Affiliations:** Department Integrative Zoology, University of Vienna, Althanstraße 14, 1090 Vienna, Austria

**Keywords:** Lophophorata, Epistome evolution, Coelomic cavities, Phylactolaemata, Lophopodidae, Forked canal

## Abstract

Phylactolaemate bryozoans are the sister-group to all remaining bryozoan taxa. Consequently, their study is essential to reveal and analyze ancestral traits of Phylactolaemata and Bryozoa in general. They are the only bryozoans to possess an epistome which traditionally has been regarded as shared with phoronids and brachiopods. Contrary to older observations, an epistome was recently reported to be missing in the early branching phylactolaemate *Lophopus crystallinus*. In this study, the ontogeny of the lophophoral base and also its three-dimensional structure in adult specimens was reinvestigated to assess whether an epistome is never formed during ontogeny and absent in adult specimens. The results show that organogenesis during the budding process in this species is similar to other, previously investigated, species. The epistome anlage in *L. crystallinus* forms in early buds from the outer budding layer which penetrates the two shanks of the u-shaped gut. This ingression of the epithelium further proceeds distally and starts to wrap over the forming ganglion. The adult epistome is a rather short, but present bulge above the cerebral ganglion with prominent muscle bundles traversing its cavity. Distally it is arched by the forked canal that in *L. crystallinus* has a particularly thick and prominent epithelium in the three median tentacles. This study shows that neither during ontogeny nor in the adult stage an epistome is absent. The epistome is less pronounced than in other phylactolaemates, but otherwise similar in its general structure. Consequently, an epistome can be assumed to be present in the ground pattern of Phylactolaemata.

**Electronic supplementary material:**

The online version of this article (10.1007/s00435-018-0402-2) contains supplementary material, which is available to authorized users.

## Introduction

Phylactolaemata is a small group of exclusively freshwater inhabiting bryozoans. Its only ~ 80 species are distributed all over the globe (Massard and Geimer 2008). Six or (maybe) seven families are currently assigned to this taxon: Stephanellidae, Lophopodidae, Cristatellidae, Pectinatellidae, Plumatellidae, Fredericellidae (e.g., Okuyama et al. 2006; possibly also Tapajosellidae; Wood and Okamura 2017). Since phylactolaemates represent the sister taxon to all remaining bryozoans (Waeschenbach et al. 2012), they are important for the reconstruction of ancestors and can aid in morphological comparisons to possible sister groups. Especially, the Lophophorata concept unites Bryozoa with Phoronida and Brachiopoda into a monophyletic group (Hyman 1959) based on morphological features such as a ciliated, coelomate tentacle crown or lophophore. Another feature present in phoronids, brachiopods and phylactolaemate bryozoans is a lip-like fold over the mouth opening, the epistome (e.g., Hyman 1959). In bryozoans a coelomic cavity is present in the epistome that is in open connection to the remaining visceral coelom (Gruhl et al. 2009; Schwaha et al. 2011). Muscles are also present traversing the coelomic cavity of the epistome or lining its epithelial wall (Gawin et al. 2017). Functionally it is considered to be involved in the feeding process (Wood 2014). Classical morphological studies found this epistome in all analysed representatives of phylactolaemates (e.g., Braem 1890; Marcus 1934) with some variations on it size and musculature. Contrary to previous descriptions (Marcus 1934), an epistome was recently described to be absent in the lophopodid *Lophopus crystallinus* (Gruhl et al. 2009). Instead of an epistome, a ciliated bulge was described to be in its place.

Internal phylogeny of Phylactolaemata has shown contrary to previous interpretations that the gelatinous forms such as lophopodids or cristatellids branch off earlier than the chitinous, more attached colonial types (plumatellids and fredericellids) (Hirose et al. 2008). A recent analysis on cystid morphology and evolution confirms this view (Schwaha et al. 2016). Still, despite some incongruences in the topology of the phylogenetic tree, lophopodids are always one of the earliest branching families (Okuyama et al. 2006; Hirose et al. 2008). This implies that either lophopodids (or at least *L. crystallinus*) have lost the epistome or it was acquired as new character in non-lophopodids. Because it is important to recognize ancestral phylactolaemate and thus bryozoan features, the presence or absence of an epistome is crucial for outgroup comparisons. Accordingly, to evaluate whether an epistome is formed during ontogeny and possibly reduced in later development, budding stages and adults of *Lophopus crystallinus* were analysed to assess whether an epistome is missing in ontogeny and adults.

## Materials and methods

Specimens of *L. crystallinus* were taken from a culture at the Natural History Museum, London and fixed by A. Gruhl in 2010 and sent to the author. Fixation for sectioning was either in glutaraldehyde or paraformaldehyde–glutaraldehyde mixture. Fixed specimens were rapidly dehydrated using acidified dimethoxypropane followed by several rinses in pure acetone. Dehydrated samples were infiltrated and embedded in Agar Low Viscosity Resin (Agar Scientific, Stansted, Essex, UK). Cured blocks were sectioned on a Leica UC6 ultramicrotome (Leica Microsystems, Wetzlar Germany) at 1 µm thickness. Sections were stained with toluidine blue. Serial sections of budding stages and adult lophophoral bases were documented and analyzed either with a Nikon E800 or NiU light microscope with a Nikon Ri1 or Ri2 microscope camera (Nikon, Tokyo, Japan). Import, alignment and reconstruction with Amira 6.3 (FEI Visualization Sciences Group, Mérignac Cédex, France) followed basically by methods described by Ruthensteiner (2008) and Handschuh et al. (2013). Snapshots were taken with the Amira software.

## Results

### Budding stages

**Fig. 1:**
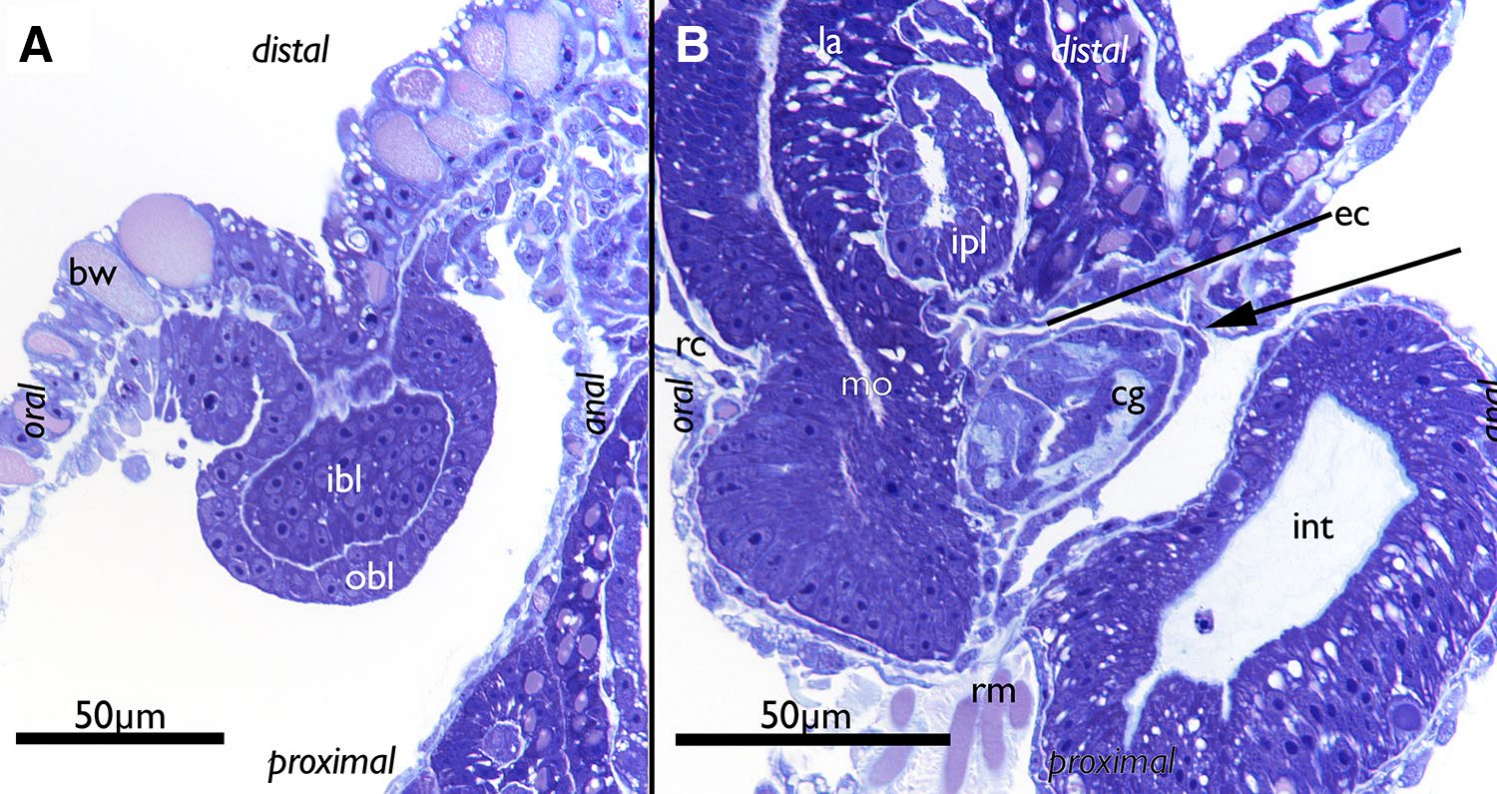
Histological details of buds of *Lophopus crystallinus*. Semithin sections, toluidine blue. **a** Section of the body wall and an early bud consisting of the outer and inner budding layer. **b** Section of a late bud with most organs differentiated showing the thin epistome coelom distally of the differentiating cerebral ganglion. The arrow points to the open connection of the epistome coelom with the remaining coelomic cavity. *bw* body wall, *cg* cerebral ganglion, *ec* epistome coelom, *ibl* inner budding layer, *int* intestine, *ipl* inner peritoneal layer of the lophophoral arm, *la* lophophoral arm, *mo* mouth opening, *obl* outer budding layer, *rc* ing canal, *rm* retractor muscle

**Fig. 2:**
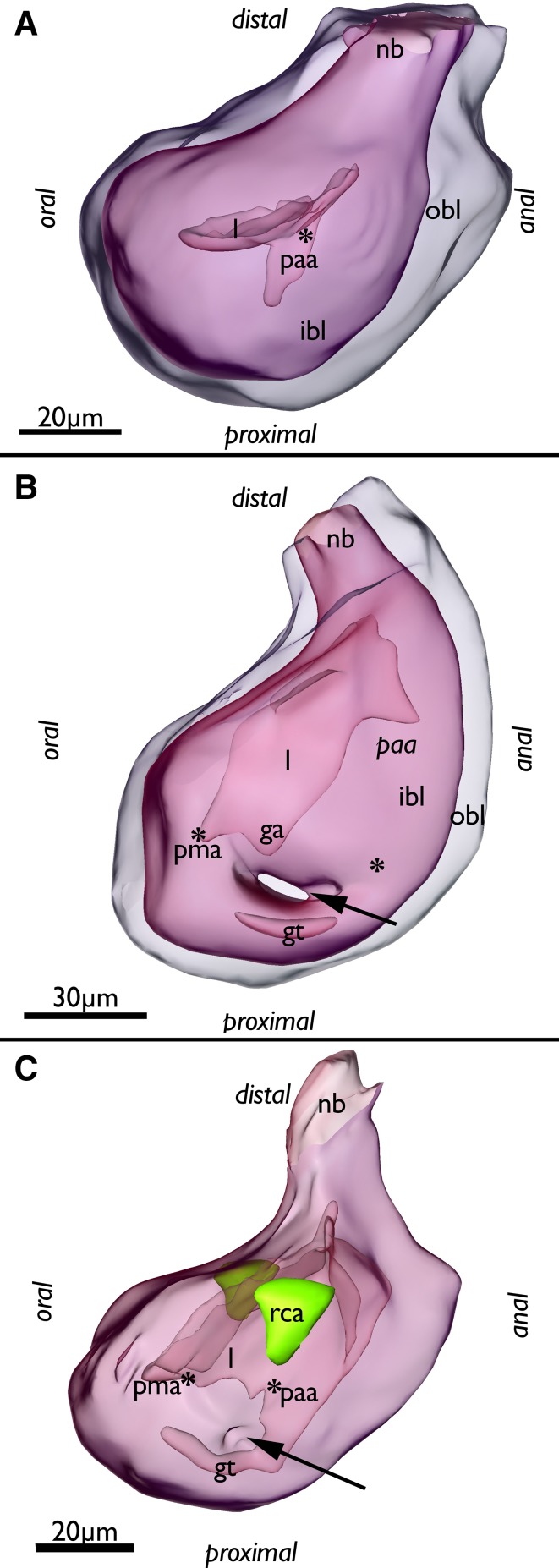
Segmentation-based 3D-reconstruction of early budding stages of *Lophopus crystallinus*. Asterisks mark areas of invaginations of the inner budding layer. **a** Early bud showing the inner and outer budding layer transparently and the differentiation of the inner budding layer. On the anal side the prospective anal anlage protrudes proximally. **b, c** Slightly advanced early budding stages. **b** Budding stage with large lumen bordered by the inner budding layer including a first shallow invagination between the prospective mouth and anal area, the ganglion anlage. Note that the outer budding layer has grown inwards separating the prospective gut parts of the oral and anal side (arrow). Also note the distinct lack the absence of a distinct lumen in the area on the anal side of the gut because the epithelium of the inner budding layer is densely packed leaving only a minute lumen. **c** Budding stage with its lumen continuous with the developing gut. Note that the outer budding layer has not grown between the prospective gut shanks, but only bulges slightly inwards (arrow). Additionally, in these budding stages lateral invaginations of the outer budding layers are evident and form distinct pockets that represent the anlagen of the ring canal. *ga* ganglion anlage, *gt* developing gut, *ibl* inner budding layer, *l* lumen of the inner budding layer, *nb* neck of the bud, *obl* outer budding layer, *paa* prospective anal area, *pma* prospective mouth area, *rca* ring canal anlage

Budding commences as an invagination of both layers of the body wall: the outer epidermis and inner peritoneum. The epidermis forms the inner budding layer, whereas the peritoneum forms the outer budding layer (Fig. 1a). The buds are always located on the frontal/distal side, situated orally of the adult zooids in a colony. Early buds are sac-shaped with a central lumen bordered by the inner budding layer (Fig. 2a). Their cells are large in comparison to the cells of the body wall and appear undifferentiated without any prominent cytoplasmic inclusions, but with distinct nuclei (Fig. 1a).

In later budding stages the inner budding layer forms two invaginations directed towards the proximal side, one on the oral and another on the anal side of the bud. The oral invagination facing the colony margin is rather shallow and represents the anlage of the foregut (pharynx, esophagus) or prospective mouth area. The anal invagination facing the adult zooids in the colony is deeper and is the anlage for the midgut and hindgut (cardia, stomach, intestine) (Fig. 2). The cerebral ganglion forms as an invagination of the orally situated floor of the inner budding layer (Fig. 2b).

**Fig. 3:**
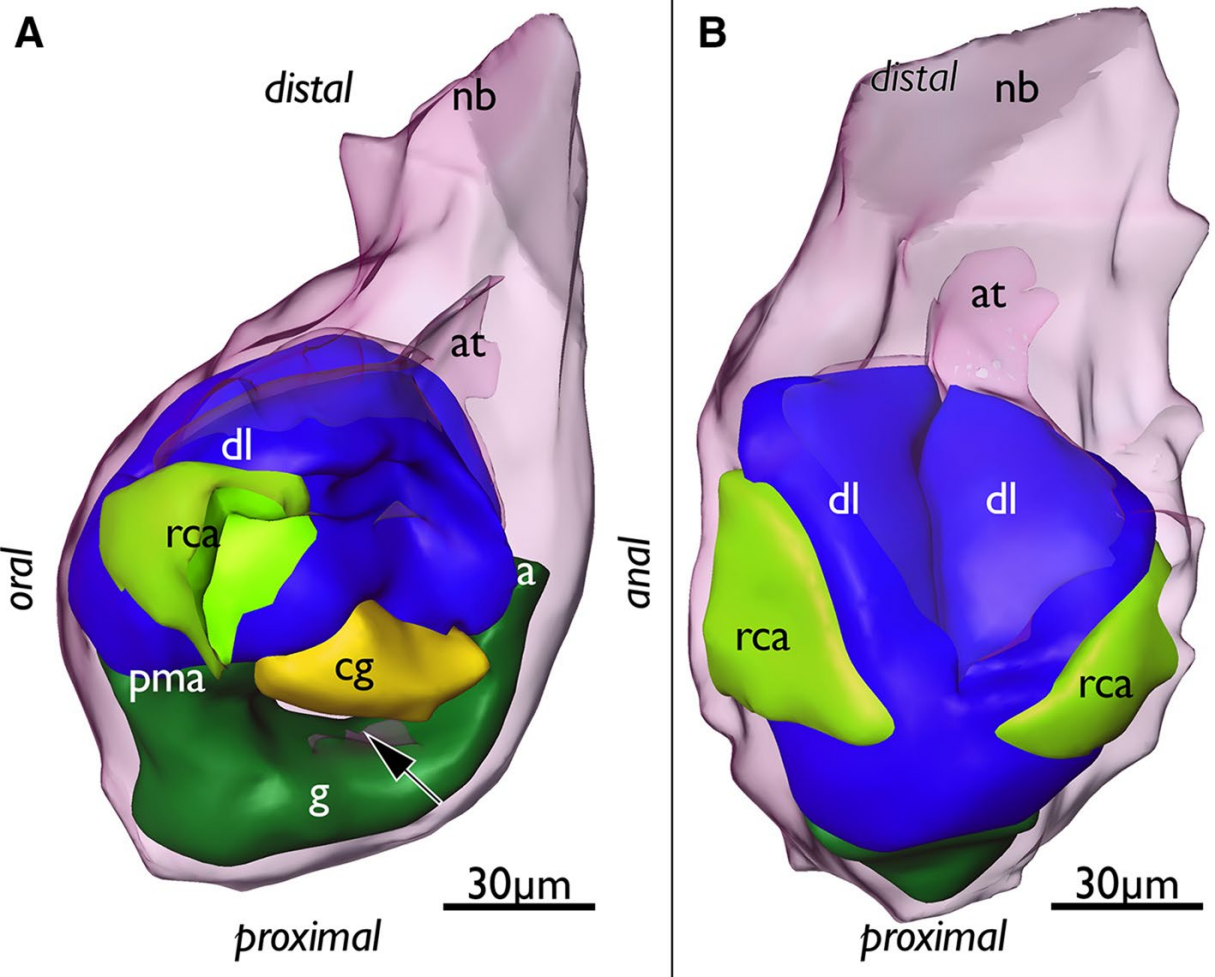
Segmentation-based 3D-reconstruction of a budding stage with differentiated anlagen of the gut, ganglion, lophophore of *Lophopus crystallinus*. **a** Lateral view of the bud with outer budding layer displayed transparently. Note that the outer budding layer separates the differentiated parts of the inner budding layer, the ganglion anlage (yellow) and the gut shanks (green) (arrow). **b** Oral view of the bud showing the two bulges of the developing lophophore and the two lateral pockets of the developing ring canal. *a* anus, *at* atrium, *cg* cerebal ganglion, *dl* developing lophophore, *g* gut, *nb* neck of the bud, *pma* prospective mouth area, *rca* ring canal anlage

**Fig. 4:**
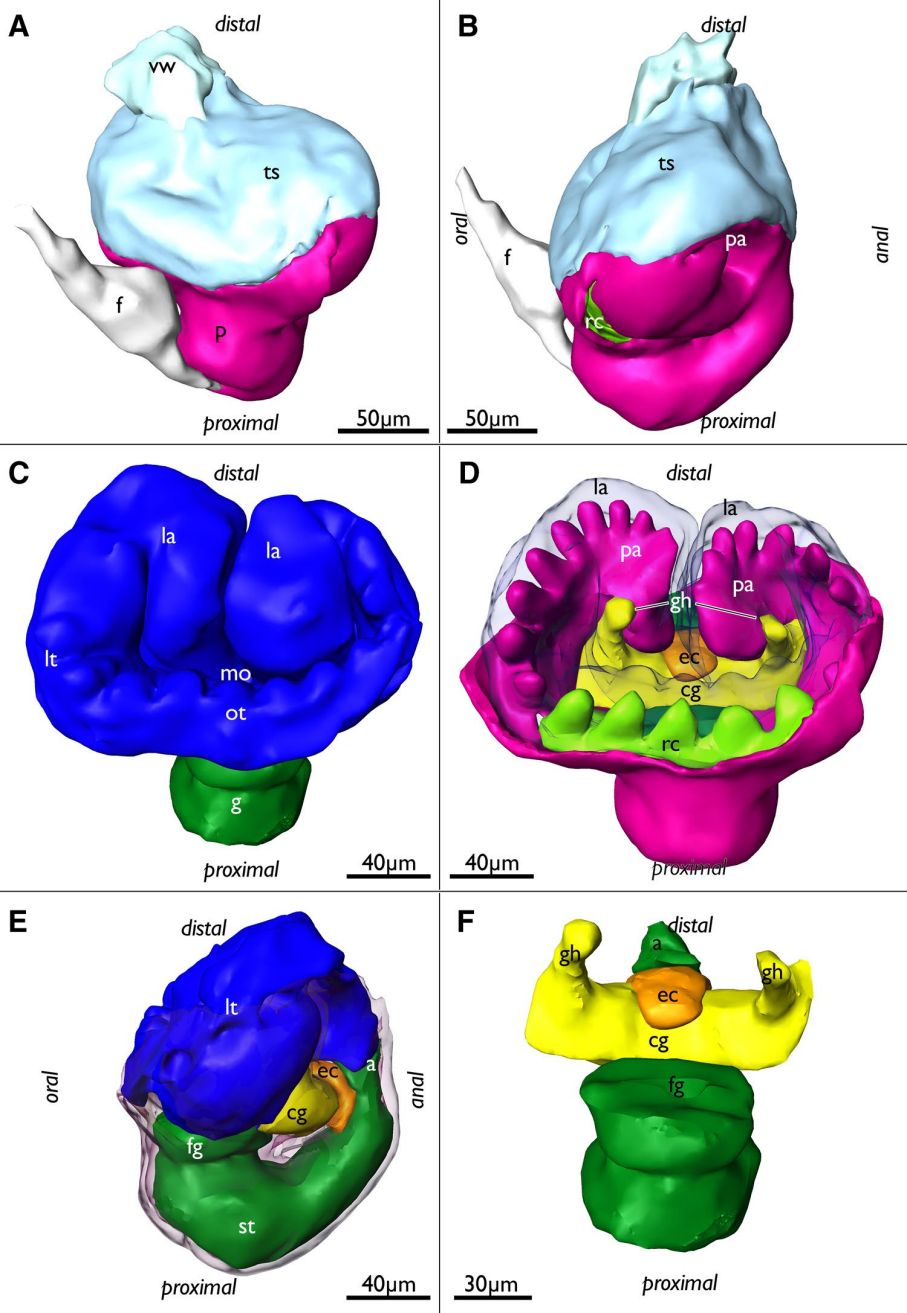
Segmentation-based 3D-reconstructions of an advanced budding stage of *Lophopus crystallinus* showing well developed tentacle anlagen of the lophophore, ganglionic horns as outgrowths of the ganglion, a complete ring canal and the epistome coelom anlage. **a** Oral view of the bud showing the outer covering peritoneal layer (pink), the tentacle sheath (bright blue) that covers the lophophore, the vestibular wall attaching the bud to the body wall, and the funicular cord (white). **b** Same as in **a**, but lateral view. Note the lateral entrances of the ring canal on the oral side (bright green). **c** Developing lophophore (blue) with anlagen of the oral and lateral tentacles and the two inner bulges, the developing lophophoral arms. Additionally, the gut (dark green) is visible. **d** Similar view as in **c**, but with the lophophore displayed transparently to show the peritoneal layer (pink) in the lophophoral arms and lateral tentacles and the complete ring canal which supplies the oral tentacles (bright green). At the base of the lophophoral base lies the cerebral ganglion (yellow) which already shows developing ganglionic horns which are distal elongations of the ganglion towards the lophophoral arms. Note that the epistome anlage (orange) protrudes medially over the ganglion in oral direction. **e** Lateral view showing the lophophore (blue), digestive tract (dark green), the cerebral ganglion (yellow) and the epistome coelom anlage which protrudes between the ganglion anlage and the anal area distally over the ganglion. **f** Oral view of the digestive tract (dark green), cerebral ganglion (yellow) and the epistome anlage medially over the ganglion. *a* anus, *cg* cerebral ganglion, *ec* epistome coelom, *f* funiculus, *fg* foregut, *g* gut, *gh* ganglionic horns, *la* lophophoral arm, *lt* lateral tentacles, *mo* mouth opening, *ot* oral tentacles, *p* peritoneum, *pa* peritoneum in the lophophoral arms, *rc* ring canal, *st* stomach, *ts* tentacle sheath, *vw* vestibular wall

Afterwards, the outer (peritoneal) budding layer starts to form three distinct paired invaginations: the first pair is located between the shanks of the gut as folds from both lateral sides which medially fuse (Figs. 2b, c, 3a, black arrows), the second pair is more distally located as two large lateral folds that push medially and represent the anlagen of the prospective lophophoral arms/bulges (Fig. 3 *dl*, b, d *pa*). The third pair of invaginations is located in the middle of both lateral sides and forms small thick epithelial pockets directed into oral direction (Figs. 2c, 3 *rca*). These represent the anlagen of the ring canal—the coelomic canal supplying the oral tentacles. The timing the anlagen differentiate seems to vary in early budding stages, because early budding stages with different degrees of differentiation were found (compare Fig. 2b, c). Later in development, these anlagen form pockets that grow larger in size and protrude orally (Fig. 3) which finally fuse in development to a single canal that remains in open connection to the remaining body cavity (Fig. 4b, d).

After these initial differentiations of the inner and outer budding layer, the main organs, i.e., digestive tract, nervous system, lophophore, are well distinguishable (Figs. 3, 4). The invaginations of the inner budding layer at the prospective mouth and anal area have fused at the future area of the cardiac valve and form a continuous gut. The lophophore anlage has two distinct lateral bulges on each side that for the most part will differentiate into the lophophoral arms. The ganglion folds from the inner budding layer and extends proximally into the area between the fore- and hindgut (Fig. 3).

In the next budding stage mainly further differentiation of the lophophore is evident. It is laterally wider, shows distinct lophophoral arms and the anlagen of individual tentacles on the oral side on the ring canal, and on the lateral sides of the lophophore (Fig. 4c, e). The underlying peritoneal layer in the developing lophophore shows more clearly the number of tentacles forming. Medially in the two inner sides of the lophophoral arms, no tentacles have differentiated yet (Fig. 4d). The lophophore is covered by the forming tentacle sheath which consists of thin layers of the inner and outer budding layer and cover the whole developing lophophore. Distally the tentacle sheath continues via the neck of the bud into the body wall (Fig. 4a, b). At the lophophoral base, the cerebral ganglion has formed two outgrowths on each lateral side (the ganglionic horns) that grow into distal direction and follow the traverse of the lophophoral arms (Fig. 4d, f). The peritoneal layer that protruded in between the fore—and hindgut in earlier budding stages has grown distally and medially passes over the cerebral ganglion into oral direction. This short coelomic extension represents the anlage of the epistome coelom (Figs. 1b, 4d–f).

**Fig. 5:**
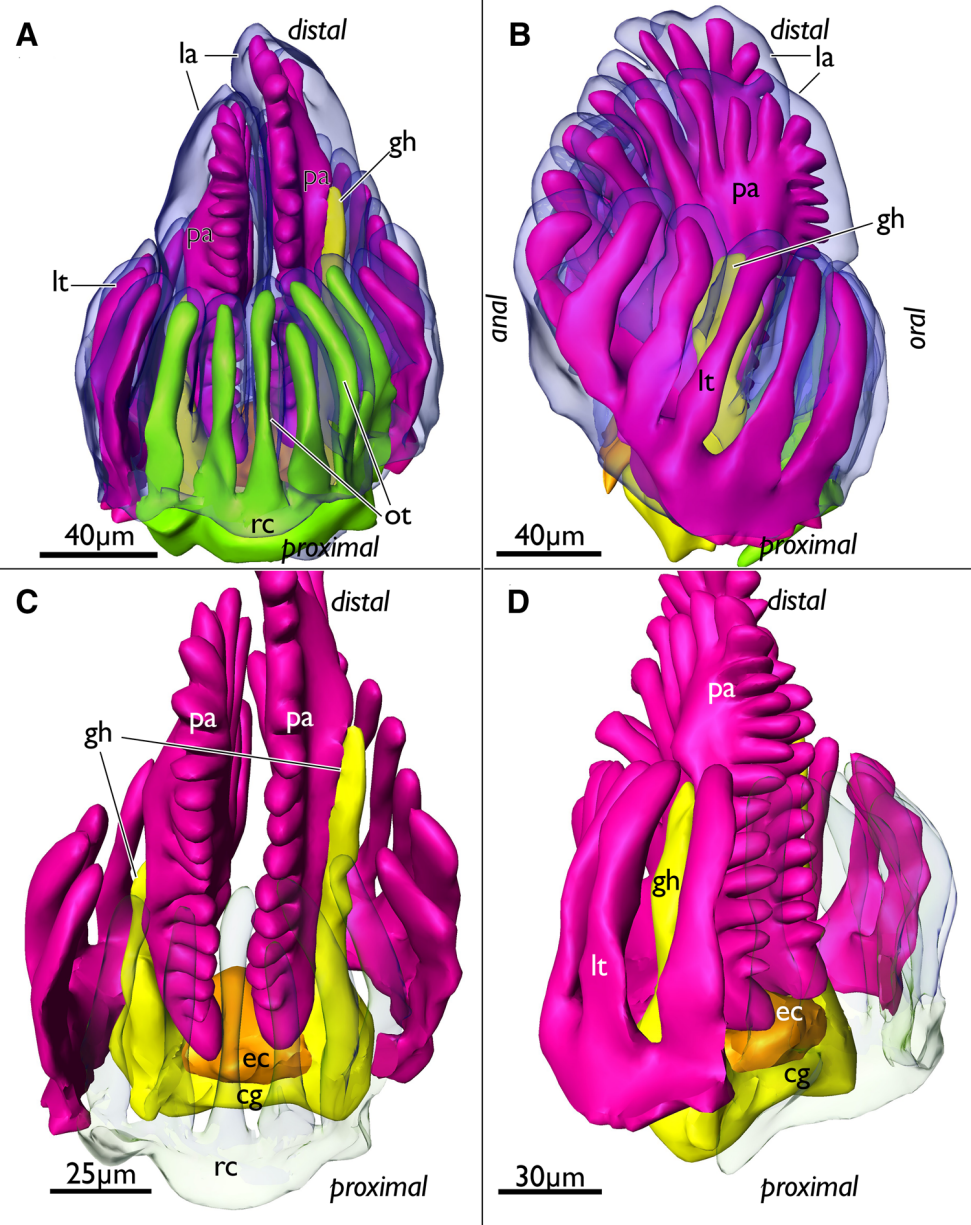
Segmentation-based 3D-reconstruction of a very late budding stage of *Lophopus crystallinus* with enlarged lophophore, ganglionic horns and epistome coelom. **a** Oral view showing the enlarged lophophore with longer tentacles. Epidermal layer of the lophophore (blue) displayed transparent. **b** Similar as in **a**, but lateral view. Note the smooth line of the epidermal layer of the lophophore whereas the peritoneal layer (pink) shows already developing tentacles. **c** Oral view of the inner lophophoral cavity, peritoneal layer (pink), ganglion (yellow) and epistome coelom (orange). Oral tentacles and ring canal displayed transparent. **d** Similar display as in **c** but more oblique view showing the epistome coelom over the ganglion. *cg* cerebral ganglion, *ec* epistome coelom, *gh* ganglionic horns, *la* lophophoral arms, *lt* lateral tentacles, *ot* oral tentacles, *pa* peritoneal layer of the lophophoral arms, *rc* ring canal

More advanced budding stages are characterized by a general size increase, which is most prominently seen in the enlarged lophophore (Fig. 5). The lophophoral arms including the ganglionic horns have grown in distal direction. Similar to previous budding stages the epidermal layer of the lophophoral arms shows only little differentiation of individual tentacles, whereas the peritoneal layer shows more distinct tentacle anlagen (Fig. 5a, b). Tentacle anlagen of the lophophoral arms have grown in number, but are in general rather short. In contrast, the oral and lateral tentacles have enlarged (Fig. 5). The epistome coelom has only slightly grown in size and broadened on its terminal end above the cerebral ganglion. It slightly protrudes towards the mouth opening (Fig. 5c, d). Distally the epistome coelom is bordered by the inner proximal area of the lophophoral arms which have not yet fused medially (to form the forked canal, see below). Additionally, no tentacles are differentiated yet in this region (Fig. 5c, d).

### Adult lophophoral base

**Fig. 6:**
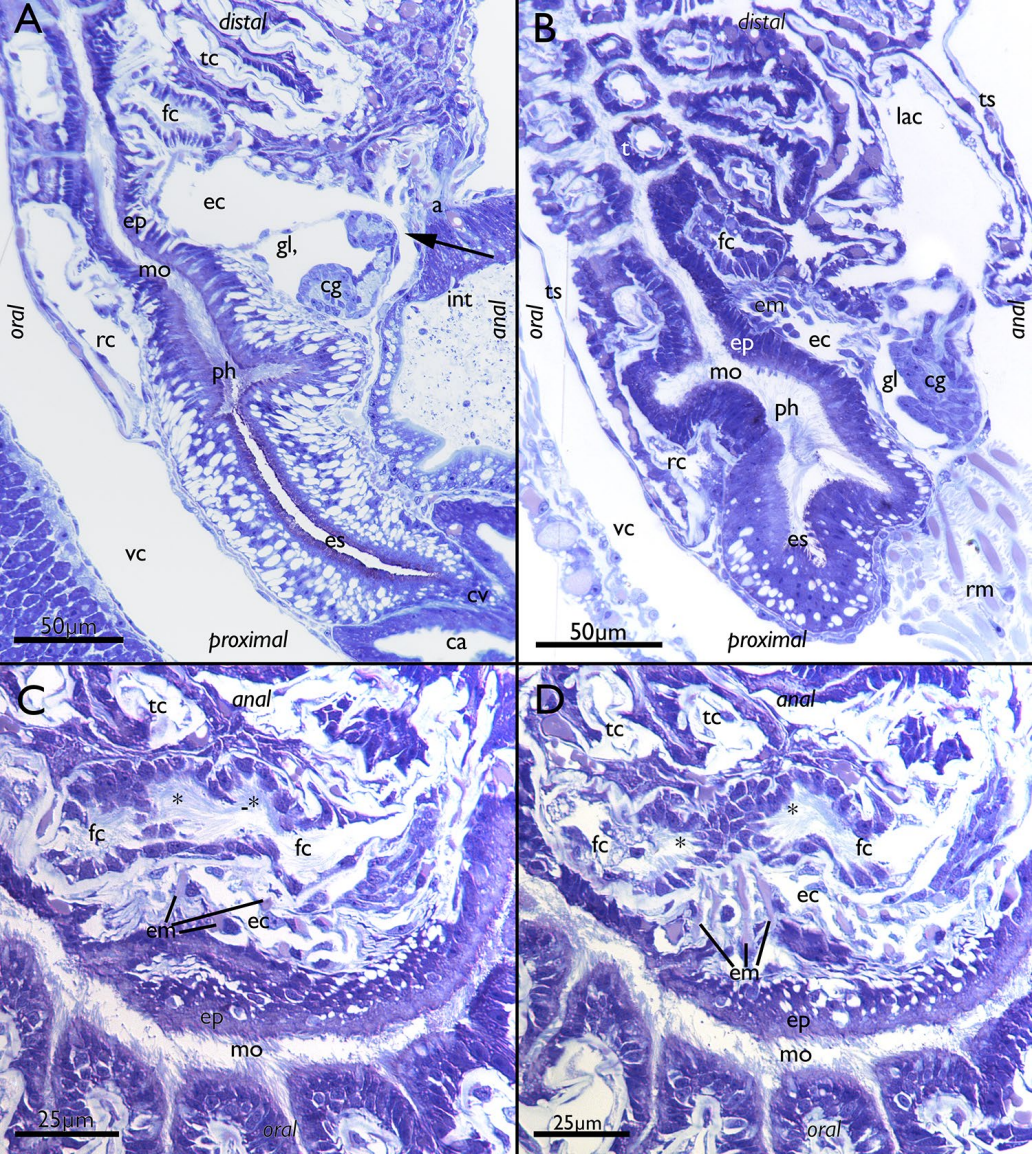
Histological details of the lophophoral base of adult *Lophopus crystallinus*. Semithin sections, toluidine blue staining. **a, b** Longitudinal sections; **c, d** cross-sections. **a** Section through the foregut with pharynx, esophagus until the cardia valve. Next to the foregut lies the cerebral ganglion with an extensive ganglionic lumen. Anally of the ganglion the peritoneum passes distally as epistomial canal into the epistome coelom. The arrow points to the open connection of the epistome coelom with the remaining coelom. Note also the distinct thick epithelium of the forked canal above the epistome coelom. **b** Similar as in **a**, but showing distinct muscle bundles in the distal epistome coelom. **c** Section through the median junction of the forked canal. Note the thickened epithelium of the forked canal and the distinct ciliation inside (asterisk). **d** Similar as in **c**, but showing the musculature through the epistome coelom. Asterisk mark the ciliation of the forked canal. *a* anus, *ca* cardia, *cg* cerebral ganglion, *cv* cardiac valve, *ec* epistome coelom, *em* epistome muscles, *ep* epistome, *es* esophagus, *fc* forked canal, *gl* ganglion lumen, *int* intestine, *lac* lophophoral arm coelom, *mo* mouth opening, *ph* pharynx, *rc* ring canal, *rm* retractor muscles, *tc* tentacle coelom, *ts* tentacle sheath, *vc* visceral coelom

**Fig. 7:**
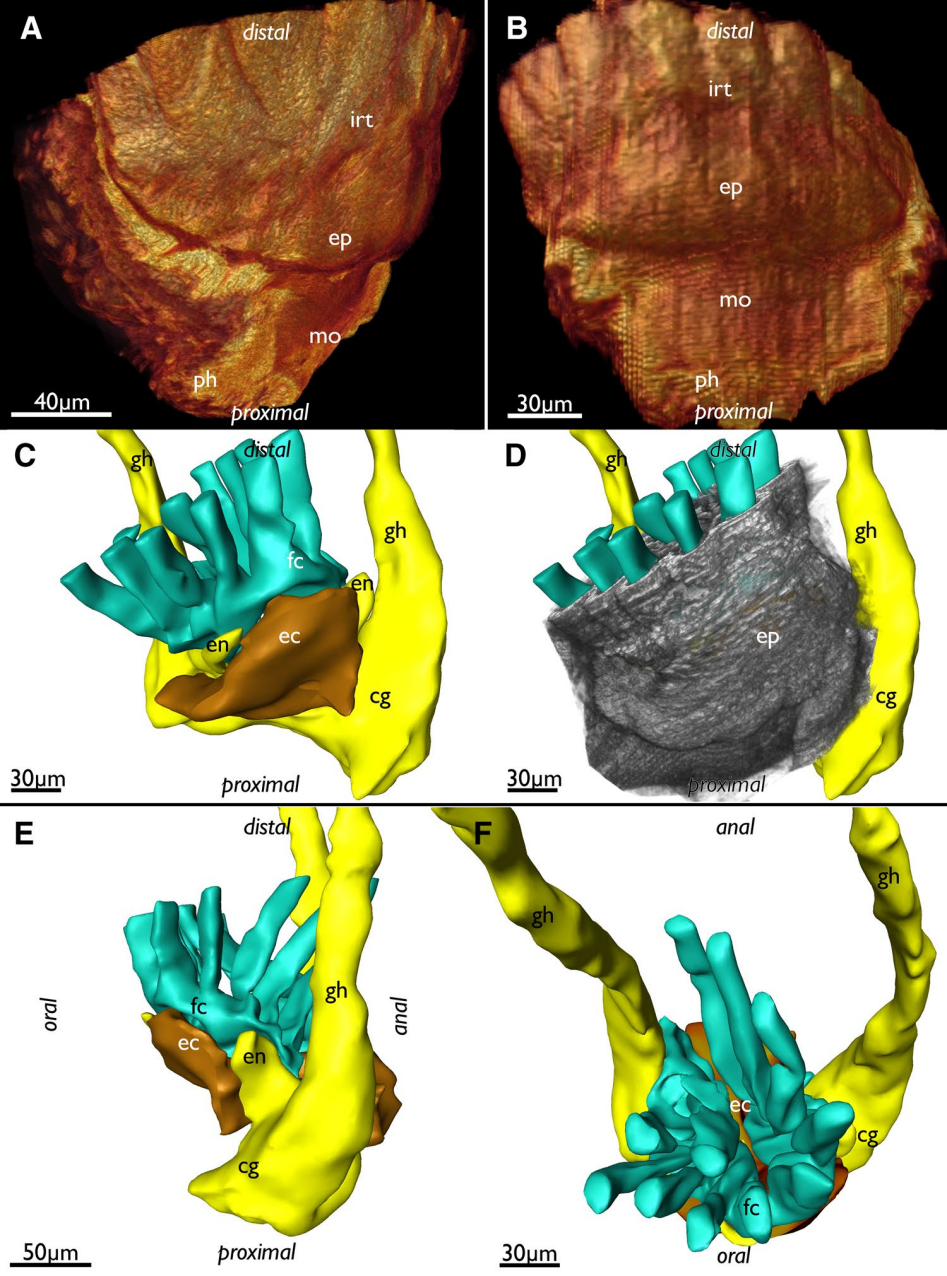
Adult lophophoral base of *Lophopus crystallinus*. **a, b** Volume renderings of the epistome area. Different views of the lophophoral base showing the epistome as a small bulge over the mouth opening directly below the inner row of tentacle within the lophophoral concavity. **c–f** Segmentation-based 3D-reconstruction. **c** View from the oral side of the epistome coelom (brown), cerebral ganglion with the ganglionic horns (yellow) and the forked canal (turquoise). Note that the lophophoral base is slightly bent to one lateral side. **d** Same image as in **c** but with the epidermal layer of the epistome displayed as grey volume rendering over the surfaces. **e** Lateral view of the surfaces of the nervous system, forked canal and epistome coelom. Note the connection of the epistome coelom with the remaining coelom on the anal side. **f** Distal view on the inner row of lophophoral tentacles on the forked canal. *cg* cerebral ganglion, *ec* epistome coelom, *en* epistomial neurite bundle, *ep* epistome, *fc* forked canal, *gh* ganglionic horn, *irt* inner row of tentacles in the lophophoral concavity, *mo* mouth opening, *ph* pharynx

**Fig. 8:**
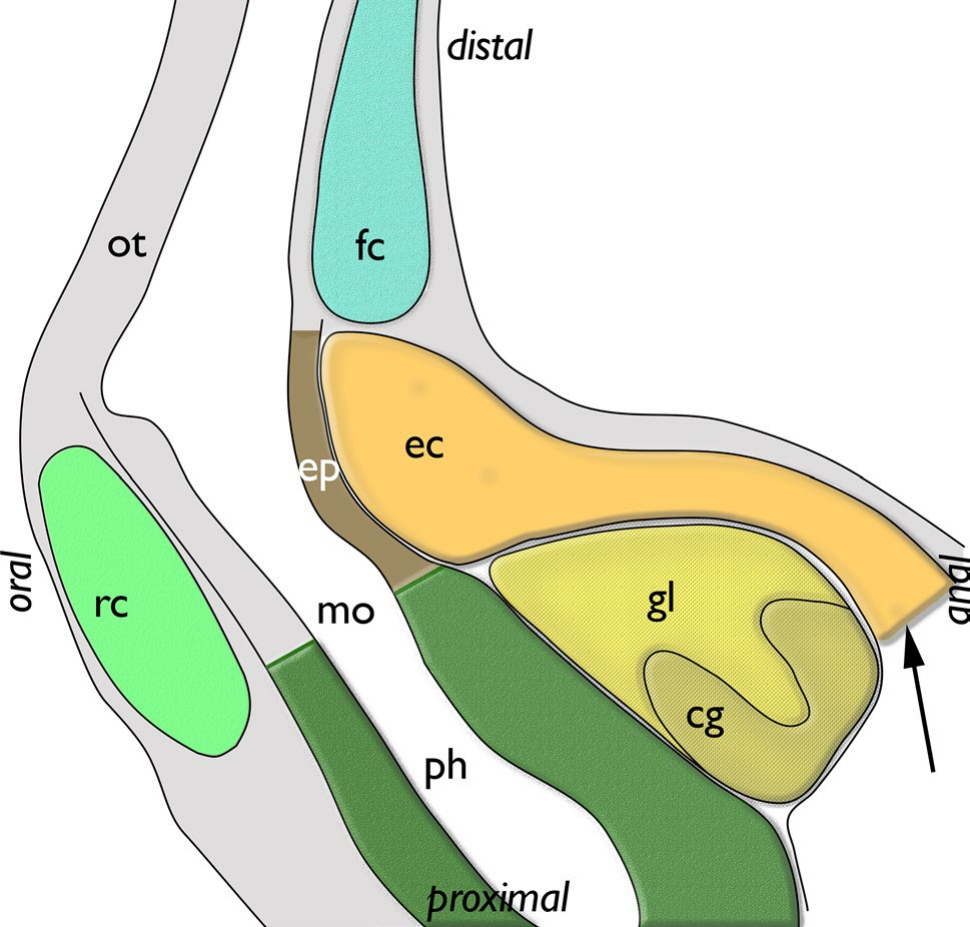
Schematic longitudinal section of the lophophoral base of *Lophopus crystallinus* showing the coelomic canals and epistome. The colors of the different structures are identical to the ones in the 3D reconstructions. The arrow points to the open connection of the epistome coelom with the remaining coelom. *cg* cerebral ganglion, *ec* epistome coelom, *ep* epistome, *fc* forked canal, *gl* ganglion lumen, *int* intestine, *mo* mouth opening, *ot* oral tentacles, *ph* pharynx, *rc* ring canal

The lophophoral base of adult zooids is similar to that of late budding stages. The cerebral ganglion adjacent to the pharyngeal epithelium is conspicuously large and contains a very large internal lumen (Fig. 6a) that also extends into the ganglionic horns. Anally of the ganglion remains the extension of the visceral coelom into the epistome (Figs. 6a, 7e). The latter is a small ciliated bulge over the mouth opening approximately 200 µm wide (Figs. 6a, c, d, 7a, b, d, 8). Its protrusion over the mouth opening is minimal. It is supplied with a broad coelomic cavity that is proximally bordered by the large area of the ganglionic lumen and distally almost entirely bordered by the arc of the forked canal (Figs. 7c–f, 8). The epistome cavity is traversed by a series of transversal muscle fibres (Figs. 6b–d, 7c, e). Laterally of the visceral epistomial coelomic canal, the two forked canals extend medio-distally in oral direction to form the coelomic supply of the inner tentacles in the lophophoral concavity (Figs. 6, 7). The forked canal opens widely into the remaining body cavity. Particularly in the three median tentacles above the epistome, the epithelium of the forked canal is conspicuously thick when compared to the remaining coelomic epithelium (Fig. 6). Distinct cilia are recognizable at the proximal openings and more distinctly in the thicker-walled parts of the forked canal (Fig. 6c, d).

## Discussion

### Budding process

The budding process including organogenesis in lophopodids such as *L. crystallinus* is poorly investigated. Only few data on the budding process in germinating statoblasts of the lophopodid *Asajirella gelatinosa* are currently available (Oka 1891). The polypide formation during budding and statoblast germination is identical (Mukai 1982). Clear differences from the current study on *Lophopus crystallinus* to that on *A. gelatinosa* are present in the ontogeny of the gut. The latter does not primarily form from an outgrowth of the prospective mouth region as indicated for *A. gelatinosa* (Oka 1891), but from the prospective anal area which was also considered by Mukai (1982). This confirms that the formation of the gut is similar if not identical in all Phylactolaemata and Bryozoa (Schwaha et al. 2011; Schwaha and Wood 2011).

Most other descriptions on the polypide organogenesis in Phylactolaemata were conducted on *Cristatella mucedo* (Braem 1890; Davenport 1890; Schwaha et al. 2011) or focus on early bud formation in plumatellids (see Brien 1960; Mukai 1982, summarized in Schwaha et al. 2011). Consequently, most of our available information on the organogenesis resides with the description of *C. mucedo* (cf. Schwaha et al. 2011). The current investigation shows that the general budding process in the lophopodid *L. crystallinus* is very similar to other phylactolaemates and in particular organogenesis is similar to *C. mucedo*. Buds in all bryozoans develop as two layered invaginations of the body wall that form the outer and inner budding layer (Schwaha et al. 2011). The outer derives and corresponds to the peritoneal layer whereas the inner to the epidermis. The peritoneal layer protrudes medially between the ‘u’ of the forming gut and lateral protrusions on the oral and lateral sides indicating the first formation of the lophophore and its arms. In the prospective mouth area, the inner budding layer forms an invagination to from the future ganglion which in early buds occupies most of the space between the ‘u’ of the forming gut. An open connection of the lumen remains that later closes. Distinct variations in the timing of certain developmental processes could be observed in *L. crystallinus* where an early bud had the anlage of the ring canal, but the peritoneal layer not penetrating between the ‘u’ of the gut and vice versa. Likewise, the anlage of the ganglion seems to vary (this study). Previous comparisons have shown that there are differences in the temporal schedule when different organs are formed in phylactolaemates (Schwaha et al. 2011). Intraspecific differences as encountered in *L. crystallinus* were so far not documented.

Ring canal formation is also similar in *Cristatella* and *Lophopus*. In both species the peritoneum slides medially from both lateral sides of the oral side which later fuse medially (Schwaha et al. 2011, this study). Generally, the anlagen of the ring canal appear earlier in *L. crystallinus* compared to *C. mucedo* and also are more prominent in the former. Lateral bulges of both budding layers directed medially and distally form the lophophoral arms. The lophophoral arms are formed earlier in *C. mucedo* and are also much more pronounced in earlier budding stages when compared to *L. crystallinus*. It appears that different parts, i.e., lophophoral arms, ring canal, of the lophophore are formed at different stages in the two species. A particular feature that is present during the budding of both *Cristatella mucedo* and *Pectinatella magnifica* is a median connection of the lophophoral arms (Schwaha et al. 2011). Possibly this character could be correlated with an earlier formation of the lophophoral arms anlage. In general, these two species also have a higher amount of tentacles (Lacourt 1968) and at least in *C. mucedo*, buds grow a very large lophophore with the lophophoral arms folded to one side (Schwaha et al. 2011). This is not the case in *L. crystallinus* where the lophophoral arms do not have any foldings.

The epistome anlage develops from the peritoneal layers between the gut shanks that consequently grow distally and arch over the developing large cerebral ganglion. The situation of the general anlage of *L. crystallinus* and *C. mucedo* is thus identical. Additionally, the later stage shows distinct similarities between the species (Schwaha et al. 2011, this study). The epistome coelom further protrudes orally towards the mouth opening. Likewise, the condition in *C. mucedo* shows that the medial coelomic extensions of the lateral inner lophophoral arms remain unconnected in most budding stages. In later development they medially fuse to form the forked canal which is present in all phylactolaemate families (Braem 1890; Oka 1895a, b; Marcus 1934, 1941; Rogick 1937; Gruhl et al. 2009).

### Adult lophophoral base

*Lophopus crystallinus* has only a very small bulge over the mouth that represents the epistome. This confirms previous descriptions (Marcus 1934), whereas most other phylactolaemates possess a more pronounced and distinct protruding lip-like structure (cf. Wood 1983; Mukai et al. 1997; Gruhl et al. 2009; Schwaha et al. 2011, 2016). The extent and distinct size of the epistome in the two other lophopodid genera, *Lophopodella* and *Asajirella*, is only superficially described (Oka 1891 for *Asajirella*; Rogick 1937 for *Lophopodella*) and appears tongue-like. However, details are not available and would require a new investigation to assess whether the small epistome is apomorphic for *L. crystallinus* or is shared among the whole family. The function of the epistome remains ambiguously discussed, but it is probably involved in the feeding process (e.g., Gruhl et al. 2009; Wood 2014). The different extent of its size, small as in *L. crystallinus* or large like, e.g., in *Cristatella* would have implications on the feeding process. The feeding process has not been studied in detail in *L. crystallinus*.

The epistome in adults contains an epistomial (coelomic) cavity that does not represent a separate coelomic cavity, but is connected with the remaining visceral coelom by a thin epistomial canal that enters the epistome between the oral and anal branch of the gut. This condition is identical in all other Bryozoa (Braem 1890; Mukai et al. 1997; Gruhl et al. 2009; Schwaha et al. 2011). This contradicts previous reports that *L. crystallinus* is lacking an epistome (Gruhl et al. 2009) and thus supports the notion that an epistome is present in the ground pattern of all Phylactolaemata.

Concerning the musculature of the epistome two main patterns have been reported in Phylactolaemata: Either bundles traverse the coelomic cavity as found in *L. crystallinus* (this study), *Lophopodella carteri* (Rogick 1937), *Asajirella gelatinosa* (Oka 1891), *Pectinatella magnifica* (Gawin et al. 2017) or muscles are only embedded in its epithelial linings as in *Plumatella* sp. and *Fredericella sultana* (Schwaha and Wanninger 2012) or *Cristatella mucedo* (Gawin et al. 2017). A mix of both systems was also reported in *Hyalinella punctata* (Gawin et al. 2017). Along with the data on *Lophopodella* and *Asajirella*, this study confirms that the first pattern with transversal muscles through the epistomal cavity is probably characteristic for lophopodids. Still, with the lack of a proper phylogeny of Phylactolaemata, it remains difficult to assess which type is ancestral for Phylactolaemata. From the latest trees (Hirose et al. 2008) it would appear parsimonious that the first pattern is also ancestral, but data on several key groups such as the stephanellids, which commonly represent the earliest branch in Phylactolaemata, are still missing.

The forked canal represents a phylactolaemate specific coelomic canal of the arc of tentacles above the epistome (Braem 1890; Gruhl et al. 2009). In the lophopodids (*L. crystallinus* Marcus 1934, this study; *Lophopodella*; Rogick 1937; *Asajirella*; Oka 1891, 1895a, b) it supplies an uneven number of tentacles that are located above the epistome. There seems to be common pattern in the three lophopodid genera that the three median tentacles possess a distinct thickened epithelial lining including distinct and abundant of ciliation. Dense ciliation has been also reported in other Phylactolaemata, but mostly on the proximal opening of the forked canal towards the remaining visceral coelom (Gruhl et al. 2009; Schwaha et al. 2011). Functionally, the forked canal was sometimes referred to a vestigial nephridium (e.g., Cori 1890, 1893), which was, however, rejected by other authors (e.g., Braem 1890). Nonetheless, the ciliation functions in transport of substances specifically to the median tentacles. Coelomocytes and sperm were considered or reported to be transported by this ciliation (Braem 1890; Oka 1985b).

The ganglion in all Phylactolaemata always contains a lumen which is epithelially lined (Gruhl and Bartolomaeus 2008). The lumen which also extends into the ganglionic horns is moderate to small in most analyzed species (Gruhl and Bartolomaeus 2008; Schwaha et al. 2011). As shown in the present study, the lumen is very large in *L. crystallinus*, contrary to previous observations (Marcus 1934). However, a large ganglionic lumen was also shown in the lophopodids *Lophopodella carteri* (Rogick 1937) and *Asajirella gelatinosa* (Oka 1895a, b). Possibly, it might be a synapomorphy of this family.

## Conclusions

The present study confirms the presence of an epistome in *L. crystallinus* and clarifies the contradicting descriptions of Marcus (1934) and Gruhl et al. (2009). Along with data from the other representatives (Oka 1891; Rogick 1937), the epistome can be considered to be present in all members of the Lophopodidae. Consequently, the body cavity situation is similar among all phylactolaemates and an epistome is also part of the ground pattern in Phylactolaemata. Furthermore, it should be emphasized that all parts of the coelomic system of phylactolaemates are confluent, and distinct, separate cavities arranged in a trimeric proximo-distal direction are not present.

## Electronic supplementary material

Below is the link to the electronic supplementary material.

Supplementary video 1: Rotation of the 3D reconstruction of the latest budding stage of *Lophopus crystallinus* showing the ring canal (light green), forked canal (turquoise), epistome coelom (orange), the cerebral ganglion and ganglionic horns (yellow) and the peritoneal layer of the lophophoral arms and lateral tentacles (pink). The general outline of the lophophore is displayed transparently (MPG 22035 KB)

Supplementary video 2: Rotation of the 3D reconstruction of the same budding stage as in supplementary video 1 but without the ring canal and the transparent lophophore outline (MPG 17435 KB)
